# Knowledge and practices related to plague persistence in plague-endemic foci, Mbulu District, Tanzania

**DOI:** 10.1371/journal.pntd.0012202

**Published:** 2024-05-30

**Authors:** Stella T. Kessy, Alfan A. Rija

**Affiliations:** 1 Department of Wildlife Management, Sokoine University of Agriculture, Morogoro, Tanzania; 2 The African Centre of Excellence for Innovative Rodent Pest Management and Biosensor Technology Development (ACE IRPM&BTD), Morogoro, Tanzania; 3 School of Life Science and Bio-Engineering (LiSBE), Nelson Mandela African Institution of Science and Technology, Arusha, Tanzania; University of Texas Medical Branch, UNITED STATES

## Abstract

**Introduction:**

Plague continues to be a major public health concern in African countries. Several social practices and environmental conditions have been associated with the reoccurrence of bubonic plague, especially in places where the disease is prevalent. Therefore, it remains important to understand people knowledge, behavior and practices related to disease risks in order to identify factors that may hinder prevention and control strategies in the foci.

**Methods and results:**

A study survey of 100 households was conducted in Mbulu district to assess plague knowledge, factors that influence flea bite and measures used for rodent and flea control. Majority of participants (86%) were familiar with the plague disease and about (50%) mentioned swelling lymph nodes as a common symptom. Most of the participants (62%) claimed to observe human plague cases during the long rain season. The majority of participants (97%) reported to experience flea bite in their domestic settings, with most stating that they experienced more flea bites during the dry season. Houses with livestock had a greater likelihood of flea bite (OR = 2.7; 95% CI: 0.36–18.80, p = 0.267) compared to houses with no livestock. Furthermore, residents reported using both local and chemical methods to control rodents and flea inside houses. Most respondents preferred using local methods in flea control. Respondents stated that the efficacy of flea control methods being applied ranged from few days to several months. There was limited knowledge of the residual effects of the agricultural chemicals being used to control fleas among the surveyed community.

**Conclusion:**

Our study highlights the importance of raising awareness and adopting effective control methods for controlling fleas and lower the risk of plague transmission and other flea borne diseases in the local communities. Sensitization of the local community on the use of appropriate chemicals for flea control is urgent to avoid any potential long-term impacts of the residual effects on the health of the local communities.

## Author summary

Plague is a significant health challenge in Africa due to environmental conditions, lifestyle, and behaviors of the people living mostly in persistent areas. Understanding people’s knowledge on the environmental conditions and lifestyle practices related to plague transmissions is essential to preventing the disease spread. We assessed community understanding on plague, factors influencing flea bites, and methods used to control rodents and fleas in the plague foci to inform the development of the potential mitigation strategies for the disease. Majority of respondents were familiar with the disease; however, few were aware of its symptoms. They reported that human cases occurred during the rainy season, with frequent flea bites experienced during the dry season. Also, households keeping livestock inside sleeping houses had a higher likelihood of flea bites compared to those without livestock. Furthermore, residents reported using both local and chemical methods to control rodents and fleas with the majority preferring local methods for flea control. Respondents stated the efficacy of the flea control methods used ranged from days to months, although there was limited understanding of the long-term health effects of the chemical residuals used among the surveyed community. The study findings emphasize the importance of understanding community awareness and preventive measures in controlling the spread of plague and call for greater community awareness raising on using appropriate flea control methods. Understanding local practices and preventive methods aids the development of interventions that are safer and more acceptable to the community, ultimately leading to more effective control strategies in these rural settings.

## Introduction

Plague persistence threatens the health and economy of affected communities in low- and middle-income countries [1–3]. Since the early 2000s, there have been many reports of human plague cases associated with fleas and rodents in three major continents; Asia, America, and Africa [4]. Countries like Madagascar and the Democratic Republic of Congo in Africa, and Peru in South America represent the highest incidence of plague [5,6]. However, the occurrence of plague is not limited just to these countries. Human plague cases for instance, have been reported sporadically in some countries, such as Tanzania, particularly in the regions of Tanga, Arusha, and Manyara [7–10]. Although the frequency of plague outbreaks in the country has decreased compared to the past, the conditions still provide a conducive environment for future occurrences [10]. Moreover, an increasing association between persistence of plague and social and environmental factors has been observed not only in Tanzania [7,10], but also in other countries such as Peru, Zambia and Uganda [11–13]. This highlights the need for understanding how human practices and behaviour in their surroundings influence the continued existence of plague. This would facilitate the identification of practices and of any other social factors that may hinder the implementation of intervention approaches aimed at preventing and controlling both the host reservoirs and vectors of the plague in the foci.

Plague is an infectious disease transmitted by the flea vector and caused by the bacterium *Yersinia pestis* [14]. Plague can be transmitted to humans through two routes: indirectly via fleabites or directly by contact with infected droplets and tissues, either from rodent hosts or other hosts [14,15]. Further, the dynamics of flea vectors and rodent hosts are mostly affected by rainfall, humidity and temperature [16–18]. These conditions increase risks of plague outbreaks especially in areas with a large population of rodent and flea species that are susceptible to the disease [19,20]. In Tanzania, for instance, rodent species such as *Mastomys natalensis* and *Rattus rattus*, and flea species including *Xenopsylla cheopis*, *Xenopsylla brasilliensis* and *Dinopsylla spp* have been associated with ongoing presence and spread of the diseases [21–23]. Additionally, some flea species including *Pulex irritans* inhabit peri-domestic areas and are known to spread plague, especially in residential areas [24–26] where both social and environmental conditions are closely linked to plague incidences [27–30]. Studies conducted in Lushoto District, Tanzania, for instance, have shown several variables related to social, cultural, economic, ecological and climate to contribute to the persistence and periodic outbreak of the disease in the area [7, 31–33]. These studies altogether strongly suggest heightened health risks to human under plague persistent conditions.

Proper flea control is important in reducing the increasing likelihood of plague outbreaks. Usually, it is important to prioritize flea control before rodent control to minimize the spread risks of infected fleas and plague bacteria [34]. Insecticide dusting has been widely used and recommended as an effective control method for rodent fleas during epidemics [35]. However, there are concerns regarding the potential health risks and environmental side effects due to its toxicity [36]. To mitigate these potential health risks, alternative methods are in use such as bait station that uses a host as a target to deliver insecticide to the ectoparasites. The approach is considered as cost-effective and specifically targets ectoparasites on the host [37,38], although additional experimental studies to enhance its effectiveness are required [39]. Additional challenge encountered in flea control is the improper use of chemical insecticides that leads to flea resistance [40–42] and increased exposure of humans to the side-effects of these chemicals. These studies altogether, highlight the importance of understanding the social-cultural dynamic factors of flea and rodent control measures towards preventing plague in affected communities.

Plague dynamics and control in affected regions in Tanzania is relatively well studied. In the early stages of plague outbreaks in Lushoto Tanzania [7,22,43], several strategies were used to minimize the spread of the disease. These included eliminating fleas and rodents, giving chemotherapy to patients, providing chemoprophylaxis to anyone in contact with infected individuals, and isolating the affected localities. However, these measures did not provide expected results in response to sporadic outbreaks. Additional strategies such as environmental sanitation, house improvements, and health education for village leaders and communities in the affected areas were introduced and implemented to eliminate rodents and fleas in their surrounds leading to decreased plague incidents in the affected communities [7,8]. Further, in Mbulu District, Tanzania, occasional cases of human plague were reported [23,44] and indicated continued spread from the primary locality. Studies conducted in these subsequently affected areas found the rodent host and flea vector, and the circulation of *Y*. *pestis* in the rodent host population was closely linked [10,45]. Despite this valuable knowledge, there is yet, limited information on the methods used by residents to control flea and rodent population and peoples’ awareness about plague disease and related risks of flea bite in their surroundings remain unclear, potentially contributing to the persistence of plague in these areas. This information, when available could be used to devise the potential strategies and to prioritize the mitigation measures to curb the diseases and associated risks to humans, thereby serving lives.

In this study, we aimed to assess peoples’ knowledge and awareness on plague disease, the risk factors related to flea bite and control measures related to the spread of plague disease in the foci. We assessed the following research questions; (i) To what extent are people in the plague foci familiar with the plague disease? (ii) What household practices and/ or other factors mentioned by the respondents influence flea bites? and (iii) What local methods are mostly used for both rodent and flea control in the study area. We hypothesized that residents in the plague foci will have different levels of familiarity with the plague disease making some people more vulnerable to plague infection. We also hypothesized that majority of the households will be experiencing flea bites influenced mostly by household practices such as keeping livestock in same residence with human.

## Material and methods

### Ethics statement

The study was approved by the institutional Review Board of Sokoine University of Agriculture, Tanzania (Ref. No. SUA/DPRTC/R/186 VOL.II). Before beginning the interview, we briefly explained the purpose of the research to the participant and thereafter a written consent was collected from each participant.

### Study area

**Fig 1:**
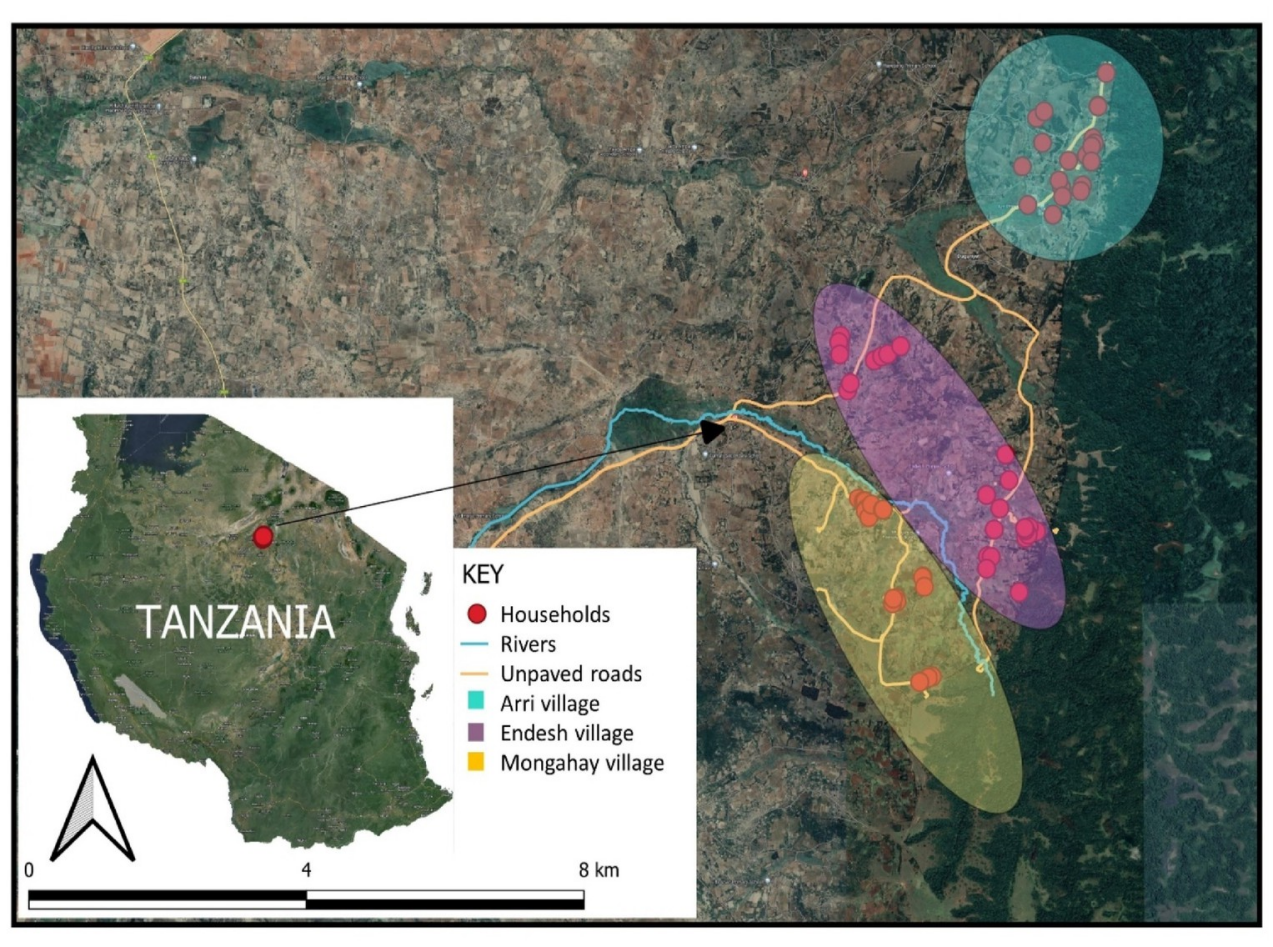
Map of Mbulu district in Tanzania showing locations of the study sites: Arri-Endeshi and Mongahay village communities surveyed. The map was generated using QGIS software (version 3.8.3 Zanzibar). Based layer sourced from Esri maps: https://esri.maps.arcgis.com/home/item.html?id=273ffd9a4c054d47843ed9642ecb143e, licensed under the Esri Master License Agreement; https://goto.arcgis.com/termsofuse/viewtermsofuse. Administrative boundaries and base layer image obtained from Administrative boundaries and the base layer image were downloaded from Open Street Map (google hybrid) https://www.nbs.go.tz/nbs/takwimu/census2012/Districts_Shapefiles_2019.zip.

The study was conducted in Mbulu district located in northern Tanzania at coordinates 03⸰ 57′ 097″S, 35° 18′ 39.60″E, with altitudes ranging from 1000 to 2400m above sea level. The study specifically focused on two villages, Arri-Endesh and Mongahay (Fig 1). The selection of this area was based on the persistence of plague outbreaks over the last 12 years, following a relatively long period of asymptomatic infection [8,10,23]. The district has a population of 238,272 with an average household size of 5.5 [44].

The climate in the study area is semi-arid to sub-humid, characterized by biannual rainfall ranging from less than 400mm to more than 1200mm. Relative humidity ranges from 55% to 75% and mean annual temperature ranges from 15 to 24°C [46] and two rainy seasons, with a long rain season from March to May and short rain season from October to December.

The residents in the area practice mixed farming cultivating short-cycle crops such as beans in December and harvested in March. The long-cycle crops such as maize and peas are planted in December and harvested in July or August. Intercropping is a common practice, with maize, beans, and peas grown together, while onions, garlic, Irish potatoes, and sweet potatoes are often grown singly. Further, livestock grazing practice varies across the two seasons: with the livestock taken outside for grazing mostly in the fallow lands, during the rainy season while animals are fed hay and stalks harvested from the farms indoors during the dry season.

**Fig 2:**
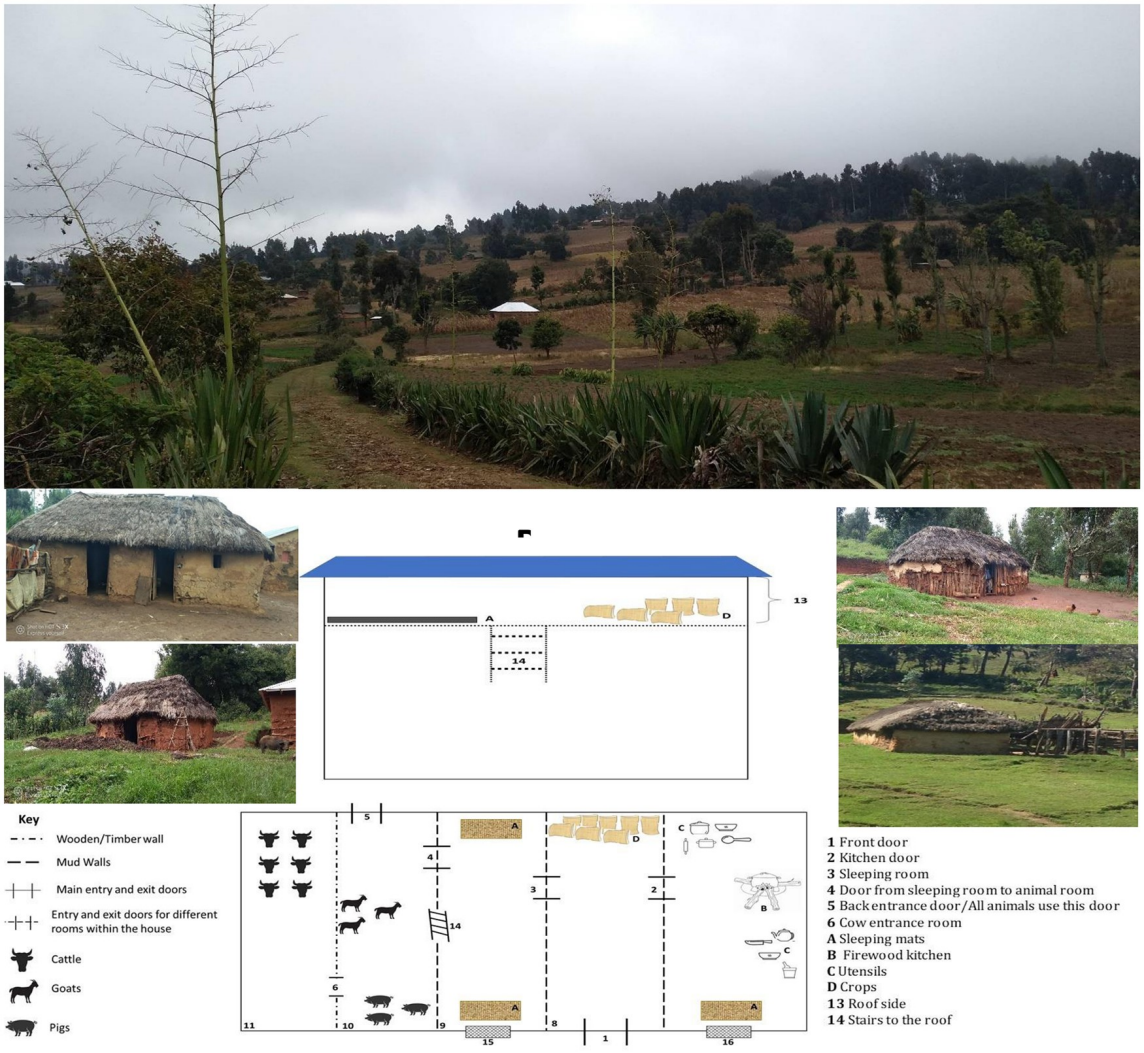
Illustration of the house design inside and outside surroundings in the study community.

Across the studied communities, majority of the human dwellings were thatched and corrugated roof with mud walls and mud floor. Most of these houses were also located near farmlands (Fig 2). Crop harvests were stored inside the sleeping houses, while livestock were kept in corrals near the sleeping and/ or inside sleeping houses. Further, sleeping practices varied, with some houses having beds with mattress or mat, while some had only one bed with mattress and several mats to sleep on the floor. Also, some households had all or one the aforementioned features and a loft chamber on the roof with a sleeping mat (Fig 2). These characteristics were recorded since they are known to influence flea infestation and survival [7,47] and so might increase the risks of flea bite inside houses and potential transmission of plague.

### Sampling design

To select houses for questionnaire, we used a list of households from the villages log books accessed from the village government office. The name of each resident was recorded in an excel spread sheet and given a unique number. Further, the RANDBETWEEN function in excel was used to randomly select houses for this survey. Mongahay village had 261 houses, 46 of which were chosen for the questionnaire survey and 64 households taken Arri-endesh (N = 531 houses). During data collection, not all household owners were present even after multiple visits perhaps were away for the farming activities so, we selected additional houses to fill the gap enabling us to survey 100 households in total. All interviews were conducted with either the head of the house or any adult household member chosen to represent the head of the house for the interview.

The questionnaire included both open and closed ended questions and aimed to understand information on the plague, the existence of fleas and rodent in their household environments, flea bites, social factors associated with the plague disease as well as the methods employed by residents to control both rodent and flea. Before beginning the interview, we briefly explained the purpose of the research to the participant and thereafter a written consent was collected from each participant. We did not ask respondents of their names during interview to ensure confidence in them and confidentiality of the data. All interviews were conducted in Kiswahili, with notes taken to ensure accuracy and reliability of the gathered data. Field data collection for this particular study was completed for 3 months.

### Data analysis

Before analysing the data, the responses from the survey were entered into a spread sheet and reviewed for errors with some coded to support some of the analysis. For instance, a question about experiencing flea bites was coded as 1 for “Yes replies” and 0 for “No replies” to create binomial response variable data. Also, questions with multiple responses were sorted based on their similarities and presented as percentage.

To assess the extent of respondents’ knowledge about plague disease in the foci, their experiences with flea bites throughout different seasons, and the methods they use to control rodents and fleas, we did a descriptive analysis. Furthermore, to assess factors that influence the probability of flea bites in the residential areas, we build a generalized linear model (GLM) implemented in the MASS package. The first model included four factors; the presence of livestock, animal corral, flea seasonality and house type as predictor variables. The response variables were the "the “yes" and "no" indicating occurrence or absence of flea bites. The relative influence of each variable in the model was evaluated by using drop 1 function, deleting non-significant model term in a backward step-wise process until the final model was reached. At each model step, the significant effect of each variable was assessed independently using the Wald test [48]. The best model fitting the data was chosen using the Akaike Information Criterion (AIC). Furthermore, a tab model function from the sjPlot package was used to generate a table summarizing the results of the best model and provide information including odds ratios, confidence intervals, p-values. A p- value less than 0.05 was considered significant. The data analysis was conducted in R version 4.3.1. The data and R codes underlying the analysis is found in DRYAD [49].

## Dryad DOI

https://datadryad.org/stash/share/iDOvCqdJs68ODhDL3g_t4GiAxwd2P3TLRXTFhb7tlw4

## Results

### Demographic information

A total of 100 residents were interviewed, of which 63% were male and 37% were female. The respondent age ranged from 19 to 91 years old. Irrespective of gender, the majority of the interviewees were between the ages of 19–60 years old (86%). Approximately 68% of the houses had a household size of six or more people, with the average household size of 7 members per household. In the study area, the majority of the respondents (85%) had primary education. Also, the majority (93%) were engaged in farming and livestock keeping, while others (7%) pursued different occupations, such as business and government employment and few were secondary school students.

### Knowledge and awareness of the plague disease among studied local community

**Fig 3:**
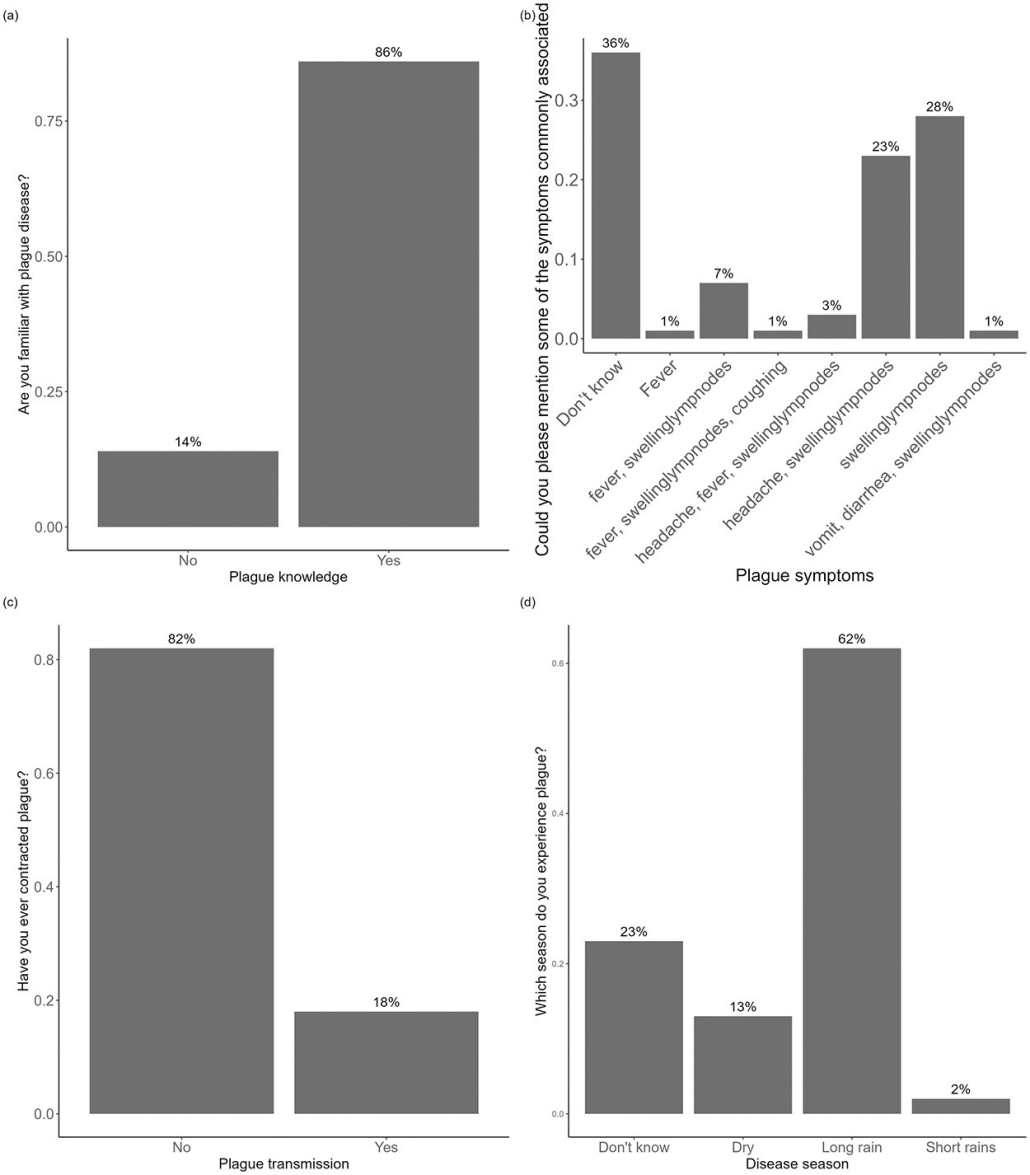
The frequency of responses in relation to different aspects of plague knowledge and experiences, (a) plague knowledge, (b) plague symptoms, (c) plague transmissions and (d) human plague cases seasonality.

The majority of respondents (86%) were aware of the plague disease (Fig 3a). However, when asked about specific symptoms of the disease, 36% were unable to provide a response. Interestingly, about 51% respondents mentioned swelling lymph nodes as a common symptom (Fig 3b). But when asked if they or any member of their household had ever been affected by plague, the majority 82% responded with NO and only 18% reported to either have experienced personal illness or the occurrence of plague within their family members (Fig 3c). Additionally, when asked about where they would initially seek assistance or medical help in the event of experiencing plague, the majority of respondents (82%) indicated a preference for hospital. However, a few respondents mentioned alternative sources of help, such as their father (1%), health officers (1%), or village leaders (6%). Moreover, the participants’ understanding of the plague’s seasonality was evident, with a majority (62%) associating human plague cases with the long rain season. In contrast, only few (2%) linked the disease to the short rainy season (Fig 3d).

### Factors that influence flea bite

**Fig 4:**
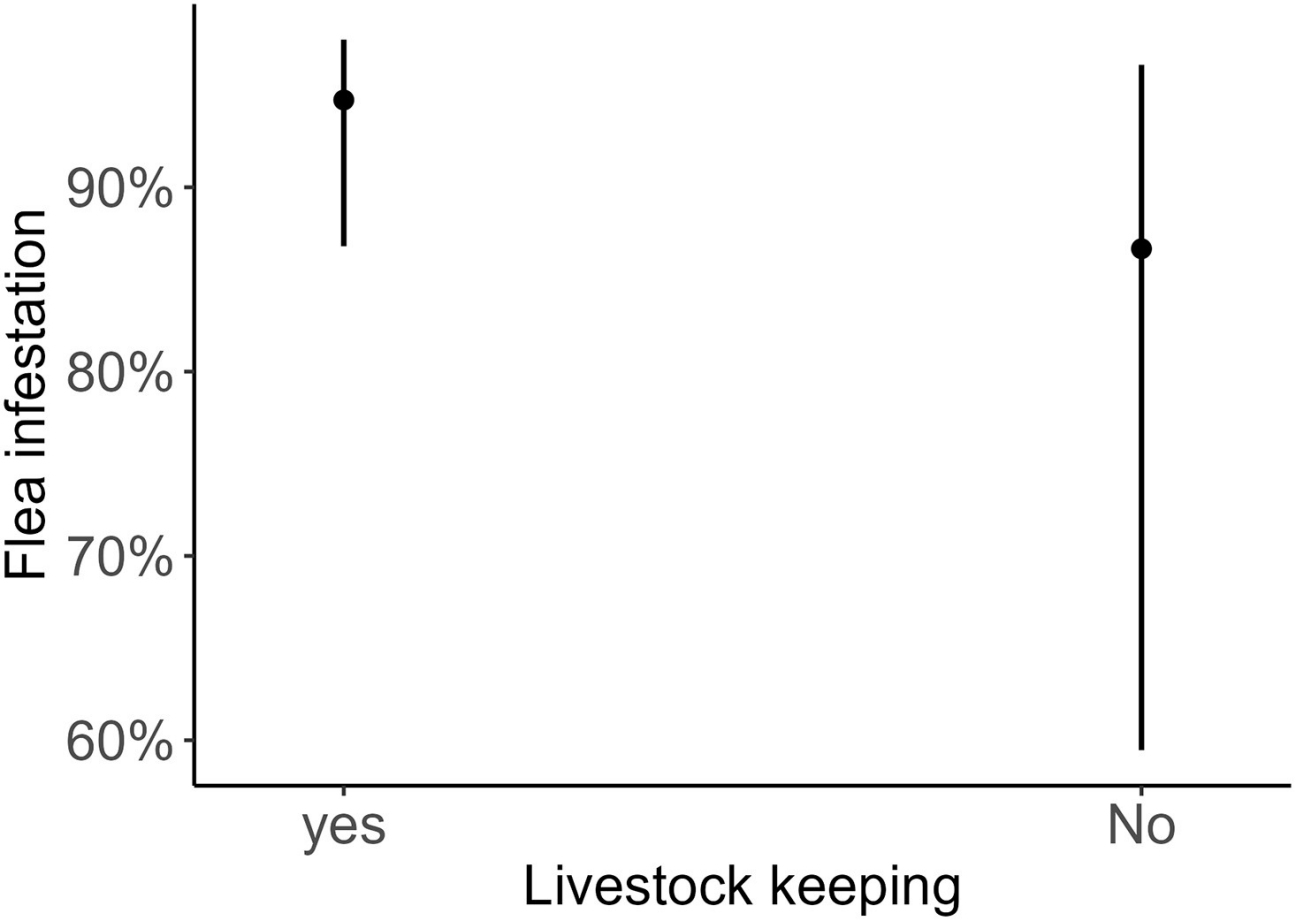
Effect predicted by generalized linear model assessing the effect of livestock keeping on probability of flea bite. The analysis revealed that, respondent who reported keeping livestock had a higher probability of experiencing flea bite compared to those who did not keep livestock.

Most participants (97%) reported experiencing flea bites in their domestic environments. When asked about the specific time of the season when they mostly get these flea bites, majority (84%) indicated the dry season. Also, our GLM results showed that residents who keep livestock have 2.77 times higher odds of experiencing flea bites than those who do not keep livestock. However, this association was not statistically significant (Table 1 and Fig 4).

**Table 1:** Odds ratio and corresponding confidence intervals and p-value from the final best-fitting Generalized Linear Model (GLM).

	Flea bite					
*Predictors*	*(No respondense)*	*Estimate (SE)*	*Odds Ratios*	*95%CI*	*Z-value*	*p-value*
(Intercept)		1.87(0.76)	6.5	1.80–41.57	2.46	**0.014**
keeplivestock [Yes]	76	1.02(0.92)	2.77	0.36–15.80	1.11	0.267
keeplivestock [No]	24					

### Methods employed by the residents for controlling rodents and fleas

**Fig 5:**
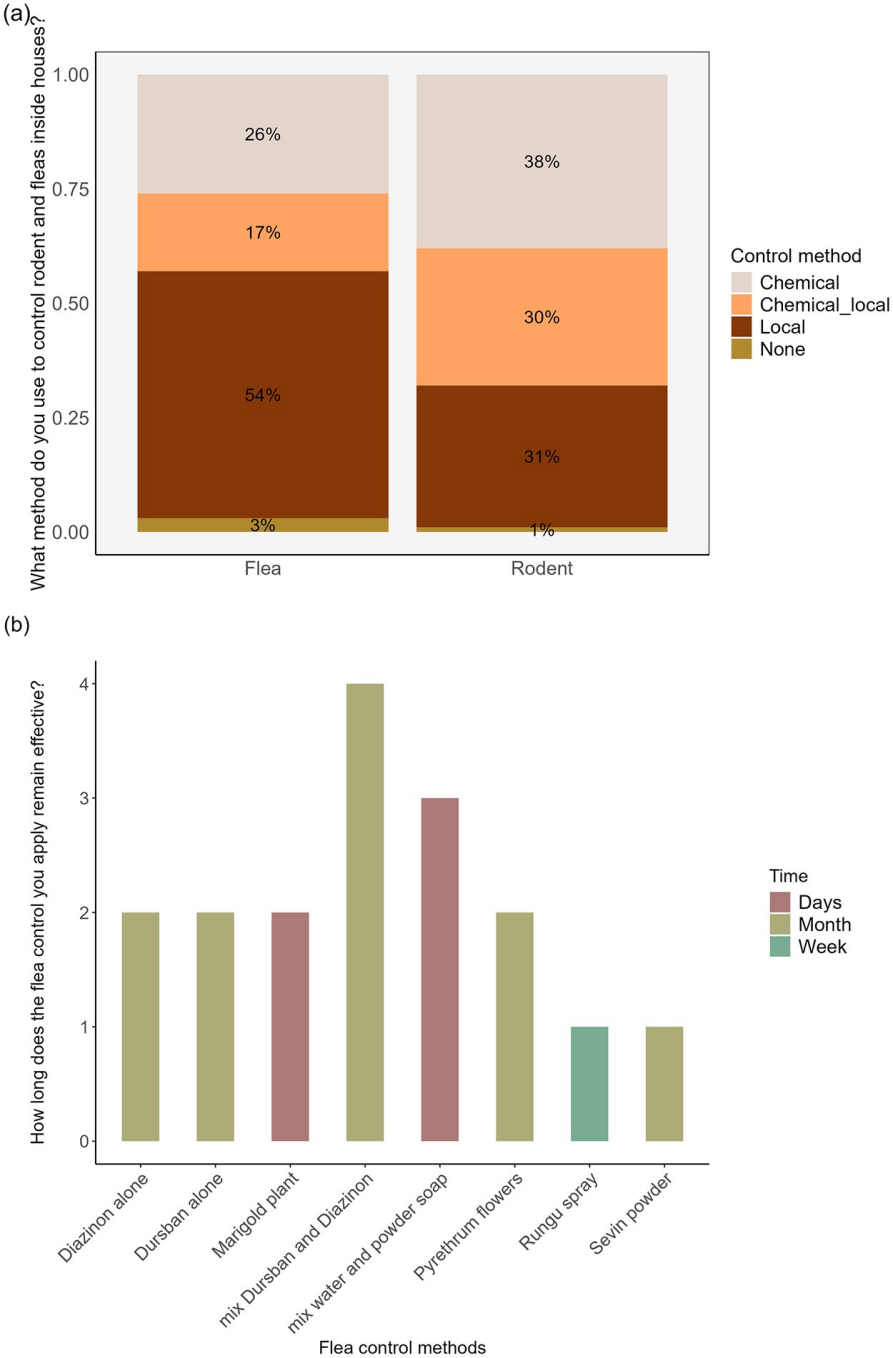
(a) Percentage of respondents using rodent and flea control methods and (b) Duration of effectiveness of flea control methods as claimed by respondents after application.

The residence in the community used a combination of chemical and natural methods to control rodents and fleas in their homes. These methods were used either separately or blended or blended natural and chemical approaches (Fig 5a). On rodent control, the application methods were relatively similar. About 38% of the response reported to be using only chemical methods, 31% relied on natural methods, and 30% of the respondents reported to employ both chemical and natural methods. The chemical methods used for rodent control specifically involved the use of rodenticide, while the local methods included the presence of cat and baited traps.

In flea control, the majority of the respondents (54%) relied on local methods (such as sprinkling water on the floor and spreading pyrethrum flowers inside house), followed by 26% who preferred chemical methods (26%). A small proportion of the respondents (17%) reported using a combination of both local and chemical methods. The chemical methods used for flea control involved the use of indoor sprays, such as Rungu, (which contain carbamate and pyrethroid insecticide like propoxur and Tetramethrin respectively), and other insecticides such as Dursban, Diazinon and Servin powder (dudu dust). Further, some respondents even mentioned combining certain chemicals, such as Dursban and Diazioan. Additionally, the local-based methods used for flea control consisted of marigold plants, locally known as “Majani ya bangi- translated as resembling marijuana leaves”, pyrethrum flowers and sprinkle water on the house floor, either alone or mixed with powder soap. Furthermore, upon asked on the effectiveness of these local methods in flea control, the respondents said the impacts varied from few days to several months (Fig 5b).

## Discussion

The purpose of this study was to examine the general knowledge and awareness of the plague disease within the human population in the foci, explore factors that influence flea bite in domestic settings, and assess the commonly used methods employed by residents for rodent and flea control. We found majority of participants were aware with the plague disease and most of them claimed to observe human plague cases during the long rain season. Also, most of the participants reported to experience flea bite in their domestic settings, especially during the dry season. Presence of livestock in domestic areas was found to increase the probability of being bitten by fleas. Furthermore, residents reported to use both local and chemical methods to control rodents and flea inside houses. In flea control, majority of respondents relied on the locally-available methods, followed by chemicals and few used a combination of both. Respondents claimed that the effectiveness of flea control methods varied from few days to several months.

The majority of participants reported their awareness and familiarity with the existence of the plague disease. However, there were some gaps in specific knowledge. Although over 50% correctly mentioned swelling of lymph nodes as a common symptom, almost one-third were unable to name any symptoms. This indicates a general awareness of plague exists, but details of clinical symptoms are lacking to some residents. Inability to identify symptoms may result in delays in seeking medical attention, which can lead to higher rates of disease transmissions and potential deaths. For instance, [50] reported that early detection and immediate treatments are crucial for controlling the spread of the disease. Further, the majority of participants knew of the association between rainy season and plague cases which may likely be associated with the decrease in rodent abundance in these areas observed during this season in our previous study [51].This reduction in rodent abundance disrupts the natural reservoir dynamics, potentially increasing the likelihood of infected fleas seeking alternative hosts, such as humans or domestic animals. This increased contact between fleas and human in the absence of sufficient rodent population increases the risk of plague transmission to humans [52,53]. Almost all participants reported to have experienced flea bites in their homes, indicating greater flea-human interactions. The presence of multiple flea species increases the likelihood of plague bacteria spreading to human population [1],hence understanding these interactions and the specific species and competence as vector is crucial for informing the effective plague control and prevention measures.

The majority of the respondents reported to experience these flea bite during dry season. This is possibly due to the favourable conditions in this season which promotes flea growth and hence increase the incidence of flea bites. Flea-host-seeking behaviour is influenced by environmental cues like temperature, humidity, and host odours, all of which can be affected by climatic changes. Warmer temperatures and humidity level above 70% during the dry season, for instance, promote flea development and population growth [54,55]. Further, presence of livestock was among factors that showed higher risk of flea bites. This is because the presence of livestock enhances flea development and survival because they provide a source of blood for adult fleas to feed, shelter and nesting sites and serve as vehicle for fleas spreading to the new hosts and environments when moving between different areas or come into contact with other hosts [56,57]. Importantly, our previous study found that rodent abundance was higher in agricultural and forest habitats within the study area [51]. Since most of the surveyed households were located closer to the farm and forest habitats, this created an ideal condition for rodents and fleas to disperse between the houses and wild places. Furthermore, the existing human practices such as storing crop indoors and cattle corals around human abodes could be providing favourable sites for rodents and flea breeding facilitating more contacts with humans, thereby directly influencing persistence and repeated human plague cases in the area. Additionally, the existing poor living style such as sleeping on mats on mud floor increased risks to flea bites and potential disease transmission as has been observed in Lushoto District, Tanzania- another plague focus [7]. Despite the existing health challenges, yet local people demonstrated efforts to minimizing the negative impacts of the disease. Both chemical and locally-based methods were in use for controlling rodents and fleas in their abodes. Rat poisoning was the main chemical choice while the majority used cats as local biological control. These findings indicate that the integrated pest management practices were common in the rural communities corroborating with previous studies on the existing use of integrated pest management in rural communities [58,59]. Interestingly, majority of the respondents relied only on plant-based approaches for flea control, either spreading leaves of certain plant species on the floor and/or under the bed to repel or kill fleas indoor. Common local methods included using marigold leaves (*Tagetes erecta*), pyrethrum flowers (*Chrysanthemum cinerariifolium*) and water sprinkling to deter or minimize risks of flea bites, similar to reports from previous studies on using botanical repellents for the insect pests [60–62]. Further, the existing use of agricultural insecticide for controlling fleas has potential danger due to lack of proper protocols, guidelines or consideration of the potential health risks due to exposure to these chemicals and their residual effects in residential settings. The majority of the chemicals used were pesticides primarily applied to kill pests in the fields, with some also being sprayed inside houses. However, it is important to note that the pesticides used indoors were not specifically intended for indoor insect control, making their use unacceptable. Despite this, many respondents reported that these pesticides were helpful as they experienced a significant reduction in flea bite even after more than three months of application. This suggests that, these chemicals will remain in use for longer time in these rural communities if alternative interventions are not available. Further, unregulated chemical use may lead to flea resistant [40–42] thereby further accelerating disease persistence in the foci.

Finally, our findings have several implications on the human health in these and similar rural communities in the tropics where plague disease is still persistent. One, to improve the health of the local communities, continued efforts to control flea and reduce risks of plague transmissions are needed. This can be achieved through increasing local awareness about the clinical symptoms of plague across the village communities, instituting cleanness policy within the village communities and deterring the cultural practices that may increase the risk of flea bite in their surroundings through community workshops and educational campaigns. Second, our results suggest poor living condition of most rural communities increase their exposure to flea bites and potential disease infection, the local government should strive to engage the local communities in economic production ventures to help lift them out of poverty to afford a decent life. Improved living standards due to elevated incomes will greatly reduce the social and cultural factors exposing them to the bites and disease thereby eradicating plague in these rural communities. Third, local transmission cycle of the disease requires further study to inform the potential improvement of the currently used local control methods. Such studies should also look into the efficacy of the plant species and the appropriate use of proper insecticides used to control flea population. Use of these integrated approaches may greatly diminish the disease persistence in these rural communities.
